# Transactions T. S. M. A.

**Published:** 1885-09

**Authors:** 


### SOCIETY NOTES.

TRANSACTIONS TEXAS STATE MEDICAL ASSOCIATION.—Messrs. Lambert & Draughn, the gentlemen printers, to whom was awarded the contract for printing the Transactions of the Texas State Medical Association for 1885, have finished the task, and the Secretary will now, in a few days, mail them to members. We unhesitatingly pronounce it by far the handsomest and most creditable volume of transactions ever gotten out for the Association. In our next issue we hope to be able to give a synopsis of its contents.

